# Data on IL-6 c.-174 G>C genotype and allele frequencies in patients with coronary heart disease in dependence of cardiovascular outcome

**DOI:** 10.1016/j.dib.2016.07.020

**Published:** 2016-07-16

**Authors:** Stefan Reichert, Axel Schlitt, Ann-Christin Benten, Britt Hofmann, Hans-Günter Schaller, Susanne Schulz

**Affiliations:** a Department of Operative Dentistry and Periodontology, Martin-Luther University Halle-Wittenberg, Germany; b Department of Cardiology, Paracelsus-Harz-Clinic Bad Suderode, Quedlinburg, Germany; c Department of Internal Medicine III, Martin-Luther-University Halle-Wittenberg, Germany; d Department of Cardiothoracic Surgery, Heart Centre of the University Clinics Halle (Saale), Martin-Luther-University Halle-Wittenberg, Germany

**Keywords:** Coronary heart disease, IL-6 serum level, IL-6 polymorphism, New cardiovascular events

## Abstract

In this data article we present data on the distribution of alleles and genotypes of the interleukin (IL)-6 c.-174 G>C polymorphism (rs 1800795) in patients with coronary heart disease (CHD) in dependence of the incidence of new cardiovascular events (combined endpoint: myocardial infarction, stroke/TIA, cardiac death, death according to stroke) within three years follow-up. Moreover, we investigated putative associations between individual expression of IL-6 genotypes and IL-6 serum level. This investigation is a subanalysis of the article entitled “The Interleukin 6 c.-174 CC genotype is a predictor for new cardiovascular events in patients with coronary heart disease within three years follow-up“ (ClinicalTrials.gov identifier: NCT01045070) (Reichert et al., 2016) [1].

## **Specifications Table**

**Table:** 

Subject area	Genetics, serology, cohort study
More specific subject area	IL-6 c.-174 G>C polymorphisms, alleles, genotypes, IL-6 serum level, new cardiovascular events, coronary heart disease
Type of data	*Table, figure*
How data was acquired	*Venous EDTA-blood (IL-6 genotyping, IL-6 serum level), standardized questionnaire for acquire follow-up data*
Data format	*Analyzed*
Experimental factors	*Venous blood for genetic and serologic analyses was acquired at baseline during in-patient phase. New cardiovascular events were evaluated within three years follow-up*
Experimental features	*IL-6 genotyping was carried out with PCR-SSP*
Data source location	*Martin-Luther University Halle-Wittenberg Department for Operative Dentistry and Periodontology*
Data accessibility	*Data are within this article.*

## **Value of the data**

• Previously unreported data about the distribution of IL-6 c.-174 G>C genotypes among German patients with cardiovascular disease in relation to the incidence of new cardiovascular events
• Data may stimulate further research in order to identify new biomarkers for cardiovascular outcome [2], [3], [4].
• Data may improve the development of individual therapeutic strategies in order to prevent new adverse events in patients with coronary heart disease.

## 1 Data

**Fig. 1:**
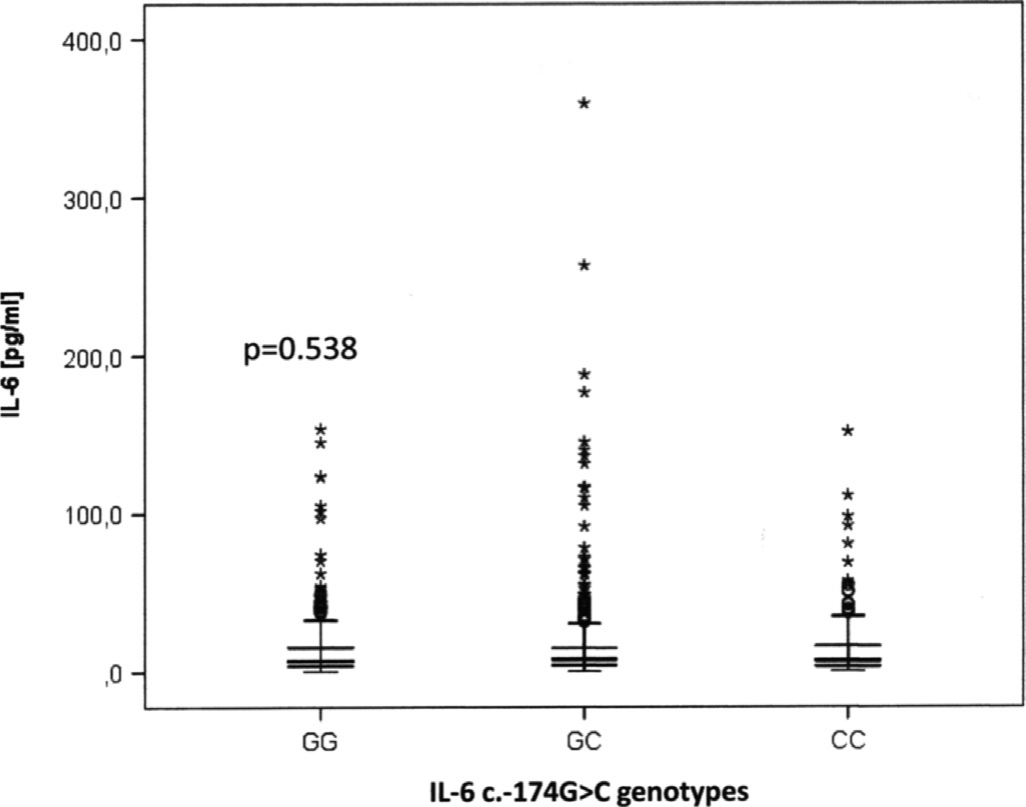
Boxplots of IL-6 serum levels in dependence of the individual expression of the IL-6 c.-174 G>C genotypes GG, CG and CC. Statistical comparison was made with Kruskal–Wallis test.

Patients who were carrier of the IL-6 c.-174 CC genotype suffered more frequently from a new cardiovascular event whereas carriers of the genotype CG experienced the combined endpoint less often. There was no significant association between the genotype GG and incidence of the combined endpoint (Table 1). Regarding the allele distribution we obtained a positive association between C allele and new adverse events (Table 2). There were no significant differences regarding IL-6 serum levels between carriers of the genotypes GG, CG, and CC (Fig. 1).

## 2 Experimental design, materials and methods

### 2.1 Patient group at baseline

The investigation was carried out in accordance with the ethical guidelines of the “Declaration of Helsinki” and its amendment in “Tokyo and Venice” and were approved by the local ethics committee.

This subanalysis comprised 942 in-patients with CHD (age 66.8±11.01 years, males 74%) at study entry from October 2009 to February 2011. Inclusion criteria were age ≥18 years and known CHD as defined by a stenosis of ≥50% of a main coronary artery by coronary angiography or percutaneous coronary intervention (PCI) or coronary artery bypass surgery (CABG). At least four own teeth except for the third molars needed to be present. Exclusion criteria were pregnancy, antibiotic therapy during the last 3 months, subgingival scaling and root planing during the last 6 months or psychological reasons rendering study participation impractical. Patients with current alcohol or drug abuse might be not completely able to understand the aim of the study and the necessity of an additional dental examination. If a drug or alcohol abuse was known from patient׳s file or a patient reported during the interview about a current drug or alcohol abuse he/she was not included in the study.

### 2.2 Follow-up

A follow-up was performed after three years from November 2013 to January 2015. The incidence of the predefined combined endpoint (combined endpoint: myocardial infarction, stroke/transient ischemic attack, cardiac death, death caused by stroke) was calculated. This information was obtained from electronic patient files, physicians, relatives, and civil registration offices. For acquiring follow-up data we sent out a standardized questionnaire. If patients did not return the questionnaires, we conducted a telephone interview with the patient or his/her relatives or contacted the patient׳s physician. If follow-up information could not be obtained from these persons, we contacted civil registration offices and requested information about current address or date of death.

From 895 of 942 initial included patients follow-up data were available (drop-out rate 4.9%) after three years follow-up (153.4±48.8 weeks). The incidence of the combined endpoint was 16.1%.

### 2.3 Determination of IL-6 serum level and c.-174 IL-6 polymorphism

Blood samples for determination of IL-6 serum level and IL-6 genotyping were taken at begin of the study from all study participants during their hospital stay. Serum level for IL-6 was determined with electrochemiluminescent immunoassay (ECLIA) using a Cobas e 602 module (Roche Diagnostics) in the central laboratory of University Clinics Halle (Saale).

The determination of IL-6 c.-174 G>C polymorphism was carried out with PCR-SSP (sequence specific oligonucleotides) using the CYTOKINE Genotyping array CTS-PCR-SSP kit (Collaborative Transplant Study, Department of Transplantation Immunology of the University Clinic of Heidelberg, Germany) in the laboratory of the Department of Operative Dentistry and Periodontology.

### 2.4 Statistical analyses

Statistical analyses were carried out using commercial available software (SPSS v.20.0 package, IBM, Chicago, IL). The IL-6 genotype and allele frequencies were calculated by direct counting and then dividing by the number of subjects to produce genotype frequency, or by the number of chromosomes to produce allele frequency. Differences between patients and controls were determined by chi-square test. The values for IL-6 serum level were checked for normal distribution using the Kolmogorov–Smirnov test and the Shapiro-Wilk test. As they were not normally distributed, comparisons in dependence of IL-6 genotypes were carried out with Kruskal–Wallis test. In general, *p* values ≤0.05 were accepted as statistically significant.

## Figures and Tables

**Table 1:** Distribution of IL-6 genotypes in dependence of the incidence of the combined endpoint (myocardial infarction, Transient Ischemic attack/stroke, cardiovascular death or death according to stroke). Statistical comparisons were made by Chi-square test. The arrow indicates the higher frequency of CC carriers among patients with new cardiovascular events.

**IL-6 174 G>C genotypes**	**All patients (n=895) %**	**No event (n=751) %**	**Event (n=144) %**	**p-Value**
CC versus	21.5	19.2	33.3↑	
GG+CG	78.5	80.8	66.7	<0.0001
CG versus	48.8	50.7	38.9	
GG +CC	51.2	49.3	61.1	0.009
GG versus	29.7	30.1	27.8	
CG+CC	70.3	69.9	72.2	0.578

**Table 2:** Distribution of IL-6 alleles in dependence of the incidence of the combined endpoint (myocardial infarction, transient ischemic attack/stroke, cardiovascular death or death according to stroke). Statistical comparisons were made by Chi-square test. The arrow indicates the higher frequency of C carriers among patients with new cardiovascular events.

**IL-6 174 G>C alleles**	**All patients chromosomes (n=1790) %**	**No event chromosomes (n=1502) %**	**Event chromosomes (n=288) %**	**p-Value**
G	54.1	55.5	47.2	
C	45.9	44.5	52.8↑	0.010
