# Supplementary material for: Data on IL-6 c.-174 G>C genotype and allele frequencies in patients with coronary heart disease in dependence of cardiovascular outcome
Source: Data Brief. 2016 Jul 16;8:1295–9. doi: 10.1016/j.dib.2016.07.020 (PMC4990635; doi:10.1016/j.dib.2016.07.020)
Supplement: Supplementary file 1 — Supplementary material [file mmc1.docx]

**Conflict of interest and source of funding statement**

The authors declare that they have no conflict of interest. The study was supported by a grant of the Deutsche Herzstiftung, Frankfurt am Main, Germany (F/34/08) and by an unrestricted grant from HAIN-Diagnostica®, Nehren, (Germany).
